# The European Union's Civil Society Strategy: a welcome milestone that now requires sustained operational support

**DOI:** 10.1093/haschl/qxag097

**Published:** 2026-04-28

**Authors:** Dheepa Rajan

**Affiliations:** European Observatory on Health Systems and Policies/WHO European Centre for Health Policy, 40 Place Victor Horta, Brussels 1060, Belgium

**Keywords:** civil society, social participation, European Union, health democracy

## Abstract

The European Commission's first Civil Society Strategy (2025) marks a significant step toward strengthening democratic resilience in the EU, with important implications for the health sector. Civil society organizations (CSOs) are central to advancing health equity, countering commercial determinants of health, and supporting evidence-informed policymaking. The Strategy's priorities—protecting civic space, ensuring sustainable funding, and enhancing participation in EU decision-making—recognize civil society as essential to effective governance. Yet, recent funding decisions in key sectoral programmes—made jointly by sectoral Directorates-General and Member States—recognize civil society as essential to effective governance. The discontinuation of operating grants in the EU4Health 2025 Work Programme, alongside reduced structural support in the LIFE Programme, weakens the independence and continuity CSOs need to contribute to public health, environmental action, and democratic processes.

While project-based funding supports valuable activities, it cannot replace the long-term functions enabled by core funding, such as policy monitoring, community engagement, and sustained dialogue with institutions. These capacities were especially visible during COVID-19 when CSOs played a crucial role in reaching underserved populations and combating misinformation. Ensuring alignment between the Civil Society Strategy and sectoral funding decisions—including reinstating operating grants—is essential for maintaining resilient health systems, advancing public interest objectives, and realizing the promise of a more participatory and equitable Europe.

Key points

- The EU's new Civil Society Strategy is ambitious, but its impact depends on aligning sectoral funding with its objectives.
- Recent cuts to operating grants in health and environment—made jointly by sectoral Directorates-General (DG) and Member States—contradict the Strategy and weaken CSOs' independence and continuity.
- Restoring stable core funding is essential for democratic resilience, effective governance, and strong public health outcomes.

The European Commission's first Civil Society Strategy, adopted in November 2025, marks an important step toward strengthening democratic resilience, or the capacity of a democratic system to maintain its core institutions and governance functions while resisting and recovering from stressors,^1^ across the European Union. Its publication is particularly relevant for the health sector, where civil society organizations (CSOs) (see Box 1) play a vital role in advancing equity, countering commercial determinants of health, and supporting evidence-informed policymaking. As part of the Commission's broader *Democracy Shield* initiative, and in line with widely accepted frameworks on co-production and participatory governance,^2–4^ the Strategy recognizes civil society as an essential component of democratic and social well-being, and is therefore welcome and timely.

Box 1. Civil society organizations—a definition Civil society organizations (CSOs) are understood here as non-state, nonprofit actors operating in the public sphere that contribute, directly or indirectly, to democratic life, including through participation, advocacy, accountability, or community representation. Not all CSOs pursue democratic participation as an explicit objective. In this paper, “CSOs” is used interchangeably with “NGOs,” as part of the broader concept of civil society.

The Strategy outlines ambitious priorities directed at EU institutions themselves: (1) safeguarding the civic space in which civil society organizations and activists operate, (2) ensuring sustainable and transparent funding for civil society organizations, and (3) enhancing the meaningful participation of civil society organizations in EU decision-making processes.

These commitments emphasize a growing understanding that civil society contributes not only to service delivery but also to the quality of governance itself. Yet for these commitments to have meaningful impact, they must be matched with the resources and mechanisms that allow European CSOs to operate independently, in the public interest, and over the long term. For health systems, this matters directly: without strong and trusted civil society partners, governments lose critical capacity for early warning, accountability, and community engagement capabilities that are foundational for resilient and equitable public health.

This is where a tension emerges. While the Strategy emphasizes the importance of sustainable funding, recent decisions in several sectoral programmes—made jointly by sectoral Directorates-General (DG) and Member States—have moved in the opposite direction. In the health sector, the EU4Health 2025 Work Programme discontinued operating grants, representing less than 1% of the total budget,^5^ that had long supported public health NGOs in maintaining expertise, engaging in policy processes, and contributing to EU-level health objectives. A similar shift occurred in the environmental field, where structural support under the LIFE Programme has been reduced. These changes risk weakening the very independence and continuity that the Civil Society Strategy seeks to promote. They also risk reducing the system's ability to respond to public health threats, as demonstrated during COVID-19, when CSOs were essential for reaching underserved populations and countering misinformation.

Operating grants have historically enabled CSOs to fulfill functions that are not easily captured within project cycles: monitoring policy implementation, participating in consultations, engaging communities, and sustaining dialogue with institutions. Organizations engaged primarily in functions such as community outreach, advocacy, accountability, and watchdog roles are thus more acutely affected (see Box 2); yet, it is precisely those kinds of activities which form the connective tissue of democratic governance. They are also essential for effective public health systems, which depend on trusted intermediaries who can mobilize communities, advocate for equitable policies, and provide early signals on emerging challenges.

Box 2. What kinds of civil society organizations receive operating grants from the European Commission? Below is a non-exhaustive list of the types of EU-level civil society organizations that have traditionally been (but are not necessarily currently being) supported through, or are eligible for, EU operating grants—mainly via Framework Partnership Agreements (FPAs).

1. *Public health & health systems NGOs*

These kinds of organizations have long received EU operating grants to support core functions such as policy analysis, coordination of national members, and EU-level dialogue.

-European Public Health Association—umbrella organization for national public health associations
-European Public Health Alliance—an alliance of national and regional public health associations, patient and disease-specific organizations, health professional organizations, and EU-level public interest health NGOs
-European Health Management Association—health systems and management network; members include health and hospital managers, healthcare providers, universities and business schools, research institutions, regional and national health authorities
-EuroHealthNet—public health promotion and health equity network, with members largely coming from national and regional public health institutes and authorities
  2. *Patient and citizen umbrella organizations*

Patient organizations, like the ones listed below, have been explicitly prioritized under EU4Health FPAs due to their role in representation, feedback, and health literacy.

-European Patients’ Forum—flagship EU patient umbrella organization
-European Cancer Patient Coalition
  3. *Disease-specific and thematic NGOs (EU level networks)*

These types of organizations traditionally relied on operating grants rather than project funding to maintain EU-level coordination and advocacy capacity.

- European Heart Network—cardiovascular disease prevention and advocacy network
- International Diabetes Federation—Europe
- European Lung Foundation
- European Brain Council
  4. *Social inclusion, harm reduction & vulnerable-population NGOs*

EU4Health operating grants have also historically supported rights-based and equity-focused health NGOs, examples of which are below:

- Correlation—European Harm Reduction Network
- European AIDS Treatment Group
- Mental Health Europe
  5. *Cross-cutting coordination structures*

Some funding went to coalitions or alliances that regrouped many of the above organizations through operating-grant-supported secretariats.

- EU4Health Civil Society Alliance—coalition of 30+ EU health NGOs whose members were FPA holders or indirect beneficiaries of operating grants

The above list demonstrates how well-resourced, well-organized CSOs can provide a public interest-based countervailing force to even better funded and better organized special interest groups, many of whom enjoy privileged access to EU policy-makers.^6–8^

Project-based funding supports valuable activities, but it is inherently time-limited and transactional. It positions civil society primarily as a service provider, rather than as a partner in governance. Contributing to a strong democracy requires continuity, independence, and the ability to engage beyond discrete projects—conditions that core funding is uniquely suited to provide.

The health and environmental sectors illustrate this clearly. Public health NGOs have been central to advancing tobacco control by providing a counter-weight to special interest lobbying through its advocacy efforts,^9^ to vaccination uptake by leveraging its community trust to counter misinformation,^10^ and to health promotion and action on non-communicable diseases through its community outreach.^11^ Environmental organizations have played a similarly important role in climate action, biodiversity protection, and pollution reduction. In both fields, civil society contributes expertise, accountability, and community engagement functions that require long-term investment. While project-based funding available through programs such as Horizon Europe or specific EU4Health calls supports valuable initiatives, it cannot replace the continuity and independence that core funding provides. Project grants are, by design, tied to predefined activities, whereas the goals of the Civil Society Strategy depend on CSOs being able to participate in agenda-setting, co-creation, and ongoing governance in the sense of substantial contribution to strategic decisions. Curtailing structural support risks weakening progress on health equity, climate action, and other public interest priorities endorsed by Member States such as the European Green Deal and the European Health Union.

The Civil Society Strategy, coordinated by the Directorate-General for Justice and Consumers, provides a strong horizontal vision—understood here as a cross-sectoral, inter-DG approach to civil society engagement across EU policy areas. Its success, however, will depend on coherence across EU institutions and programs. This will require overcoming chronic coordination challenges across DGs where a silo mentality often hinders interservice consultations, and an attitude of constructive cooperation rather than competition is required.

Strategy implementation also requires alignment between strategic commitments and sectoral funding decisions, including in areas where civil society contributions are central to public interest outcomes—those that serve broad societal needs rather than the narrower objectives of well-resourced special interest groups. While many special interest actors are far better funded and more tightly organized, they typically represent only a small minority of the population; by contrast, public interest CSOs articulate wider democratic, social, and health priorities that would otherwise be underrepresented. Ensuring that CSOs can operate independently is thus not simply a matter of budget allocation; it is a prerequisite for effective governance, public trust, and equitable health outcomes.

Delivering on this vision is a shared responsibility. Many funding decisions, including those related to operating grants, are taken jointly by the European Commission and Member States. In the case of NGO operating grant discontinuation, a recent Non-Paper issued by 13 Member States clearly laments the “timetable and consultation arrangements [which] did not allow Member States to provide input in a sufficiently timely and meaningful manner,”^12^ insinuating that a quick, intransparent procedure took place before Member States could adequately assess trade-offs and consequences.

The immediate policy ask is therefore straightforward: reinstate and stabilize operating grants in EU4Health and LIFE, address the procedural issues raised in the Non-Paper, and ensure that the Strategy's principles are embedded in upcoming Work Programs—this will require collaboration, clarity of purpose, and a recognition that civil society is an essential partner in achieving the EU's health, environmental, and social objectives. Collaboration could be better fostered through, for example, inter-DG task forces staffed with operational-level civil servants; this could also contribute to breaking the silo mentality which currently reigns during certain policy discussions. Another option to consider are long-term partnership frameworks between the EU and civil society organizations—they could anchor the Strategy's civic space and governance objectives within the time horizon it merits. Finally, defining a Strategy monitoring framework with indicators aligned with the Strategy’s objectives could serve to assign responsibility to different stakeholders for concrete implementation.

The Strategy sets out an important and ambitious direction. The next step is to ensure that the enabling conditions—sustained funding, institutional coherence, and long-term engagement—are firmly in place. With these foundations, civil society organizations can continue to contribute to a more resilient, equitable, and participatory Europe, and the promise of the Democracy Shield can be fully realized.

## Supplementary Material

qxag097_Supplementary_Data
